# Economic impact of immunoglobulin replacement therapy in secondary immunodeficiency to hematological cancer: a single center observational study

**DOI:** 10.3389/fimmu.2024.1413231

**Published:** 2024-06-26

**Authors:** Luciana del Campo Guerola, Ana Andrea García Sacristán, Antonio Portolés, Maricruz Jasso, Teresa Guerra-Galán, Eduardo de la Fuente-Munoz, María Palacios-Ortega, Miguel Fernández-Arquero, Cristina Cuesta-Mínguez, Aránzazu Rodríguez-Sanz, Ascensión Peña-Cortijo, Marta Polo, Marta Mateo Morales, Eduardo Anguita-Mandly, Teresa Benítez Jiménez, Celina Benavente Cuesta, Silvia Sánchez-Ramón

**Affiliations:** ^1^ Department of Immunology, Instituto de Medicina de Laboratorio (IML) and Instituto de Investigación Clínico San Carlos (IdISSC), Hospital Clínico San Carlos, Madrid, Spain; ^2^ Department of Immunology, Ophthalmology, and ENT, School of Medicine, Complutense University School of Medicine, Madrid, Spain; ^3^ Department of Pharmacy, Instituto de Medicina de Laboratorio (IML) and Instituto de Investigación Clínico San Carlos (IdISSC), Hospital Clínico San Carlos, Madrid, Spain; ^4^ Department of Pharmacology, Instituto de Medicina de Laboratorio (IML) and Instituto de Investigación Clínico San Carlos (IdISSC), Hospital Clínico San Carlos, Madrid, Spain; ^5^ Unidad de Investigación Clínica y Ensayos Clínicos (UICEC), Instituto de Investigacion Sanitaria Hospital Clinico San Carlos, Madrid, Spain; ^6^ Department of Hematology, Instituto de Medicina de Laboratorio (IML) and Instituto de Investigación Clínico San Carlos (IdISSC), Hospital Clínico San Carlos, Madrid, Spain; ^7^ Department of Medicine, Medical School, Complutense University (UCM), Madrid, Spain

**Keywords:** secondary immunodeficiency, immunoglobulin replacement therapy, economic impact, infections, ICU - intensive care unit

## Abstract

This is the first report of the health economic benefits derived from preventing infections through Immunoglobulin Replacement Therapy (IgRT) in patients with secondary immunodeficiency due to hematological malignancies. We conducted a retrospective population-based cohort study using patient medical history and pharmacy data from the Hospital Clínico San Carlos for 21 patients between 2011 and 2020. The pharmacoeconomic impact of using prophylactic IgRT was assessed by comparing characteristics of the SID patients 1 year before and after initiating IgRT measured by direct medical and tangible indirect costs. Results indicate a marked reduction in hospitalization days following IgRT initiation, decreasing from an average of 13.9 to 6.1 days per patient, with the elimination of ICU admissions. While emergency department visits decreased significantly, the number of routine consultations remained unchanged. Notably, absenteeism from work dropped substantially. The financial analysis revealed significant reductions in medication use and fewer ancillary tests, resulting in considerable cost savings. Specifically, total expenditure dropped from €405,088.18 pre-IgRT to €295,804.42 post-IgRT—including the cost of IgRT itself at €156,309.60. Overall, the annual savings amounted to €109,283.84, validating the cost-effectiveness of IgRT in managing SID in patients with hematological cancers.

## 1 Introduction

Antibody deficiency is characterized by either markedly low levels of immunoglobulin G (IgG) or impaired immunoglobulin functionality (1). It can originate from a primary immunodeficiency due to genetic anomalies or as a consequence of various secondary conditions such as protein-losing states, hematologic malignancies, adverse drug reactions, organ transplants, and infectious diseases. Secondary antibody deficiency (SID) is more prevalent than primary antibody deficiency, often multifactorial, associated to the underlying condition and the effects of its treatment (2). Hematologic cancers like chronic lymphocytic leukemia (CLL), lymphoma, and multiple myeloma (MM) are frequent causes of SID (2). These deficiencies can lead to recurrent pulmonary infections and increase susceptibility to viral or fungal opportunistic infections, as well as multidrug-resistant organisms (MDRO) (3). Contributing factors may also include impaired function of T cells, dendritic cells, or natural killer cells, often exacerbated by the immunosuppressive treatments administered for these malignancies (2). The repetitive nature of these infections places a significant burden on both the patient’s quality of life and healthcare resources, emphasizing the substantial economic impact of managing SID.

It has been demonstrated that intravenous immunoglobulins (IVIg) significantly reduce the incidence of severe infections in patients with CLL and hypogammaglobulinemia (3–6). Patients eligible for this therapy typically exhibit hypogammaglobulinemia (IgG <4g/L) alongside recurrent infections or an inadequate response to vaccinations (3, 5). While the cost of IVIg can be considerable—ranging from €45,000 in Germany (2018) to $108,000 annually in the USA (2018) (6, 7) —the benefits, such as reduced infection rates, fewer hospital admissions, and less need for specialized consultations and additional tests, provide substantial cost-effectiveness. For instance, a 2019 report from the Spanish National Health System noted that among the 25 most frequent causes of hospitalization, pneumonia was predominant, costing an average of €3,540.60 per stay with a typical duration of 7.7 days. Other significant diagnoses requiring hospital care included various respiratory infections, urinary infections, and sepsis (8). By decreasing infection rates, hospital stays, and other healthcare needs, the use of IVIg can potentially offset its high cost.

The primary aim of this study was to assess the economic impact of IgRT on SID. We compared the direct medical costs and work-related absences for diagnosing and treating patients with secondary antibody deficiencies for one year before and after initiating IVIg treatment, thus quantifying the overall economic benefits of this intervention.

## 2 Methods

A retrospective cohort study was conducted to evaluate the economic impact of IgRT in patients diagnosed with SID at the Hospital Clínico San Carlos (HCSC). The study included patients treated with IgRT from 2011 to 2019, with data sourced from patient medical records and pharmacy transactions. The study received approval from the hospital’s institutional research Ethics Committee (19/219-E), and all participants provided written informed consent.

The economic analysis was performed through a cost-benefit approach from the perspective of a third-party payer, using data retrospectively collected from HCSC.

We assessed the pharmacoeconomic impact of IgRT as a prophylactic measure by comparing the direct medical costs and tangible indirect costs, such as work absenteeism, for patients with SID before and after treatment initiation. These costs were assumed to be directly attributable to the immunodeficiency. However, our analysis did not include direct non-medical costs or indirect intangible costs, such as reductions in quality of life, which might also significantly affect the overall economic burden of the disease.

### 2.1 Cost determination

Costs were evaluated using the most recent social rates published for the public health service, covering a 12-month follow-up period starting from the initiation of IgRT. Costs were calculated based on component pricing using rates from official regulatory documents (B.O.C.M. Núm. 198) and the HCSC internal pharmacy price listing (in €), both as of 2019 (9). These prices were uniformly applied across all patients, regardless of treatment year, ignoring potential economic inflation or market variability.

The economic impact was analyzed by comparing costs before and after initiating treatment. Expenses not directly related to infectious diseases, such as consultations in other specialties (e.g., traumatology), were excluded unless they were a consequence of the immunodeficiency. For instance, ER visits resulting from accidents, subsequent traumatology assessments, X-rays, and rehabilitation appointments were omitted. Costs for consumables like syringes, needles, and antiseptic pads were also excluded, as they were considered consistent across both periods analyzed.

#### 2.1.1 Direct costs

The estimation of direct medical costs included the following components:

- Drug Acquisition Costs: This included costs for the main drug under study (IgRT) and any antimicrobials associated with treating infectious diseases.
- Diagnostic Procedures: Costs were associated with various diagnostic tests such as radiography (€27.23), CT scans (€190), MRI (€180), echography (€97.7), bloodwork (€130), and cultures (€2).
- Healthcare Services: This encompassed expenses related to emergency room visits, hospitalization (number of days), Intensive Care Unit stays (number of days), and outpatient clinic visits (number).
- The cost of immunoglobulin (Ig) was set at €25.25 per gram, reflecting the mean price derived from contract manufacturing prices (maquila), with the assumption that there was minimal or no drug wastage per vial. The cost of each drug was calculated based on the Defined Daily Dose (DDD) and a standard average treatment duration. For example, for azithromycin, the DDD was 0.3 grams with a typical treatment duration of three days. For all medications, during both periods assessed, it was presumed that treatments in the hospital setting were administered parenterally and those in outpatient settings were administered orally. Only patients who received IgRT at the day hospital were included in this cohort analysis.

#### 2.1.2 Indirect costs

Sick leave was accounted for patients of working age (under 65 years). Each instance that required patient interaction with healthcare services, including outpatient consultation visits, hospitalization (including ICU stays), and emergency room visits, was considered equivalent to a day of absenteeism, approximating a full working day (8 hours). The calculation of lost wages used an average hourly wage of €14.04, aligning with the salary cost per productive hour across various types of working days and activity sectors, as reported by the National Statistics Institute (10). However, additional missed workdays that were related to the patient’s immunodeficiency but did not involve hospital visits were excluded from this cost estimation.

### 2.2 Data analysis

Patient characteristics were summarized using means (± standard deviation [SD]), medians (interquartile ranges), counts, and percentages, as appropriate. Univariate generalized linear model regression with gamma distribution and log link was used to compare cost differences.

## 3 Results

We reviewed the medical records of 30 patients with SID treated between 2011 and February 2020. To eliminate potential bias related to the COVID-19 pandemic, we excluded nine patients from the study. These individuals received IgRT during the pandemic, a period characterized by quarantine and mandatory mask-wearing, which might have independently contributed to the reduced infection rates observed.

The study cohort consisted of 15 females and 6 males, with an average age of 65 years at the onset of IgRT, ranging from 35 to 85 years. All patients had hematological malignancies such as NHL, CLL, or other lymphoproliferative disorders. At the start of IgRT, all but two patients were in complete remission, a status that was maintained throughout the following year, and none had other life-threatening conditions. There were no reported deaths in the year post-IgRT initiation.

Prior to receiving IgRT, the average IgG level among these patients was 365 mg/dL, below the 400 mg/dL threshold commonly used to justify starting IgRT in cases of secondary humoral immunodeficiencies. Although three patients had IgG levels above this threshold, all demonstrated an inadequate response to vaccination, thereby meeting the criteria for IgRT.

**Figure 1:**
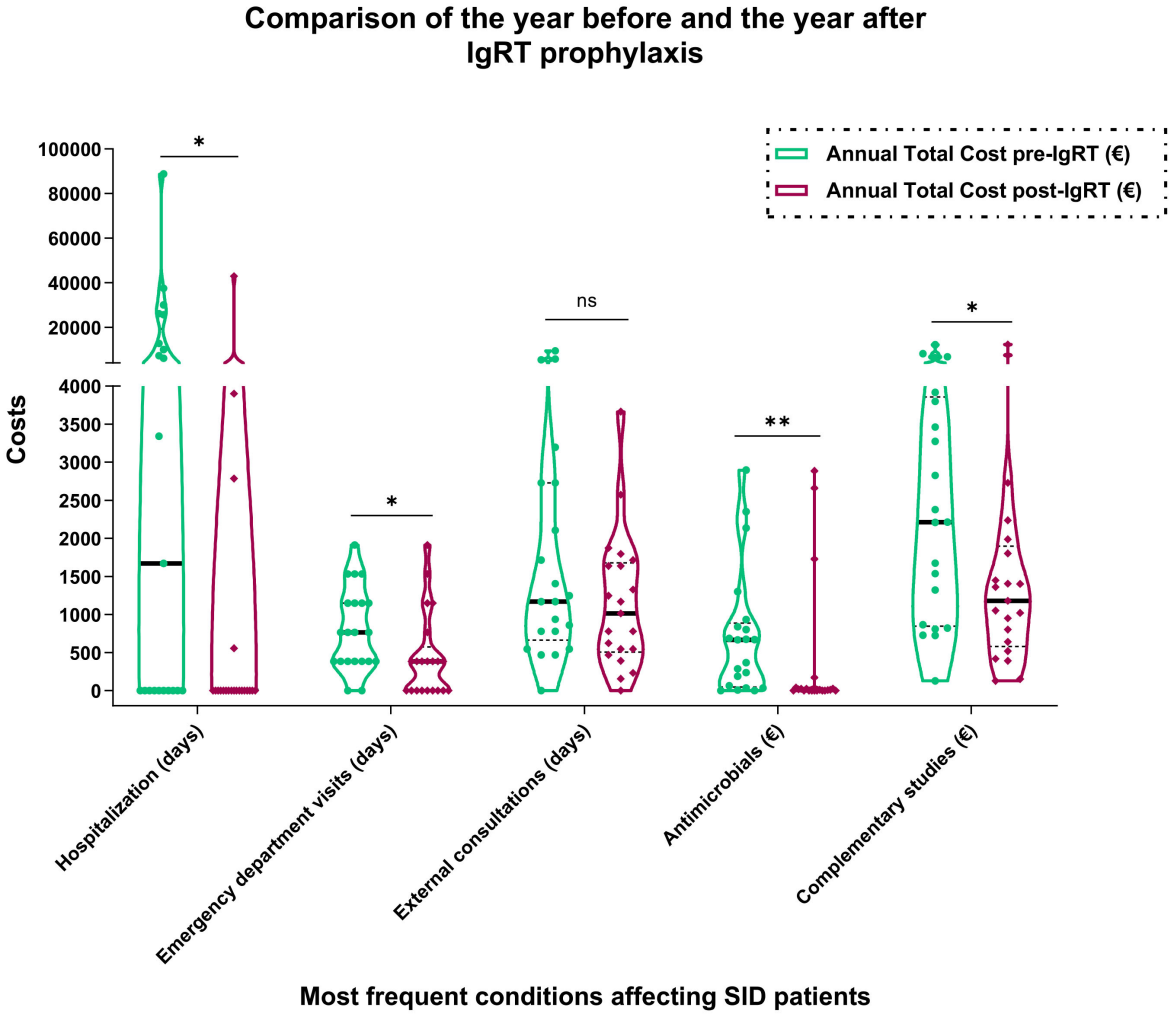
Comparison of Health Economic Costs Before and After Initiation of Immunoglobulin Replacement Therapy. *, P ≤ 0.05; **, P ≤ 0.01. ns, non-significant.

**Figure 1** and **Table 1** display detailed statistics on the total number of days patients were hospitalized due to infections, including stays in the ICU, as well as the number of emergency room (ER) visits and outpatient consultations they required. Prior to starting IgRT, the average hospitalization days per patient annually was 13.9, with an additional 2.7 days in the ICU, 2.1 emergency visits, and 26.5 consultation visits.

**Table 1:** Costs of the most frequent conditions affecting SID patients—comparison of the year before and the year after the prophylaxis with IgRT.

	Absolute No. 1 year Before IgRT (days)	Absolute No. 1 year After IgRT (days)	Annual Total Cost pre-IgRT (€)	Annual Total Cost post-IgRT (€)	Global Annual Savings between pre-IgRt and post-IgRT (€)
Direct costs					
**ICU hospitalization (number of days)**	58	0	249,389.00	50,130.00	199,259.00
**Hospitalization (number of days)**	293	129			
**Emergency department visits (number)**	44	24	16,852.00	9,192.00	7,660.00
**External/Outpatient consultations (number)**	557	310	43,446.00	24,180.00	19,266.00
Antimicrobials and others (€) *	–		15,179.20	7,604.10	7,575.10
Complementary studies (€)**	–		66,294.30	41,537.20	24,757.10
**IgRT**	–	–	0.00	156,309.60	-156,309.52
Indirect costs					
**Sick leaves (absenteeism) (days)**	124	61	13,927.68	6,851.52	7,076.16
**TOTAL**			405,088.18	295,804.42	**109,283.76**

* Others include antivirals, antifungals and erythropoiesis stimulant agents.

** Complementary studies include basic blood work, radiography, CT scans, MRI, echography and cultures. **, P ≤ 0.01; *, P ≤ 0.05.

One year after beginning IgRT, there was a notable reduction in the mean number of hospitalization days per patient to just 6.1, with this decrease proving statistically significant (p=0.0459). Moreover, patients no longer required ICU stays post-IgRT. The frequency of ER visits also dropped significantly to an average of 1.1 per patient (p=0.0152), although the change in the number of consultation visits was not statistically significant, with an average of 15.5 visits post-treatment.

Overall, the total number of hospitalization days was cut by more than half following the initiation of IgRT, completely eliminating the need for ICU admissions. Additionally, the total number of sick days decreased significantly from 124 days before IgRT to 61 days after, translating to a substantial saving of €7,076 in terms of reduced absenteeism.

Along with decreased hospitalization days and reduced absenteeism, our analysis highlighted considerable cost savings stemming from fewer medication needs (p=0.0018) and fewer additional diagnostic tests for infections (p=0.0467). Before the implementation of IgRT, the average expenditure per patient per year for the evaluated parameters was €19,289.91, culminating in a total of €405,088.18 for the entire cohort. After initiating IgRT, the annual costs per patient decreased to €14,085.92, resulting in an overall total of €295,804.42. This includes an average of €7,443 per patient per year directly attributable to IgRT costs, amounting to a total of €156,309.60 for IgRT across the cohort, with each patient receiving an average of 23 g of IgRT per month. Taking into account all associated costs—hospital stays, ER and after the initiation of IgRT, the total annual savings per patient per year amounted to €5,203.99, or €109,283.84 for the entire cohort. This significant reduction underscores the economic benefit of IgRT, in addition to its clinical efficacy in reducing the frequency and severity of infections in patients with SID.

## 4 Discussion

To our knowledge, this represents a pioneering study examining the economic implications of IgRT in SID to hematological malignancy during the first year of treatment compared to the preceding year.

Due to the underlying malignancy and following anticancer treatment, patients frequently develop a secondary antibody deficiency, predisposing them to heightened infection susceptibility and increased medical care requirements. Upon meeting the criteria such as hypogammaglobulinemia with recurrent infections and/or inadequate response to vaccination, the patients should opt for IgRT, known to effectively reduce infection rates and avoid organ damage. This study endeavors to assess the economic benefits attributable to the reduction of infections despite the high cost of IgRT.

Following initiation of IgRT, a significant reduction of hospitalizations and complete cessation of ICU admissions were observed. This led to a considerable costs savings derived from cutting down the hospital stays and decreased absenteeism. Notably, within this patient cohort, there was either, a reduction of infections, days spent hospitalized while getting treatment, or both. Of the parameters examined, savings associated with reduced hospitalization duration were the most pronounced.

The reduction in ER visits due to infections and need for outpatient consults was also noteworthy. Not only did these patients seemed to present fewer illness episodes attributed to their immunodeficiency, but they also required fewer complementary studies for diagnosis or follow up care. This could be attributed to less severe comorbidities or had fewer repercussions on their health that needed medical follow up.

A noticeable shift was observed in medication usage among these patients, with reduced expenditure on antibiotics, antivirals, antifungals, and erythropoiesis-stimulating agents post-initiation of IgRT. The study did not account the cost of material used for medication administration, nor did it assess medications such as anti-inflammatories, antipyretics, corticoids, etc.

It is important to note that several medical records documented patients expressing noticeable health benefits following IgRT administration. Furthermore, the prophylactic effect of IgRT in preventing infections could potentially enhance the effectiveness of costly anticancer drugs and advanced therapies. These treatments may be jeopardized by life-threatening infectious diseases, underscoring the critical role of infection prevention through IgRT in maintaining treatment efficacy and patient safety.

The limitations of this study are that information in patient records may not be complete, as some may have received medical care in other hospitals. Additionally, the duration of anti-cancer treatments was assumed using an average, however, they could be either suspended/changed before time or prolonged for lack of response. Direct non-medical, and indirect intangible costs were not included into the analysis.

Similar to observations in primary immunodeficiencies, while IgRT is costly, the expenses incurred due to infections are substantially greater, both in financial terms and in impact on quality of life (11, 12). Before receiving IgRT, the costs of hospitalization, ER visits, outpatient consultations, complementary tests, medicaments, and absenteeism due to the complications of SID, the cost was €405,088.18. Afterwards, it was €139,494.82, around a third of the money spent before initiating IgRT. Even taking in account the €156,309.60 that IgRT costs, there is a considerable savings. The total annual savings per patient per year amounted to €5,203.99.

In conclusion, our observational cohort study demonstrates that despite the high costs associated with IgRT, its use is economically justified for treating patients with SID who meet specific clinical and immunological criteria. This treatment significantly reduces healthcare expenses related to frequent and severe infections, thereby affirming its cost-effectiveness and advocating for its broader application in eligible patients.

## Data availability statement

The raw data supporting the conclusions of this article will be made available by the authors, without undue reservation.

## Ethics statement

The studies involving humans were approved by Scientific and Ethics Committee of the Hospital Clínico San Carlos. The studies were conducted in accordance with the local legislation and institutional requirements. The participants provided their written informed consent to participate in this study. Written informed consent was obtained from the individual(s) for the publication of any potentially identifiable images or data included in this article.

## Author contributions

LG: Conceptualization, Data curation, Formal analysis, Methodology, Validation, Writing – original draft. AS: Formal analysis, Supervision, Validation, Writing – review & editing. AP: Formal analysis, Methodology, Supervision, Validation, Writing – review & editing. MJ: Data curation, Supervision, Validation, Writing – review & editing. TG-G: Supervision, Validation, Writing – review & editing, Formal analysis, Writing – original draft. EF-M: Supervision, Validation, Writing – review & editing. MP-O: Supervision, Validation, Writing – review & editing. MF-A: Supervision, Validation, Writing – review & editing. CC-M: Supervision, Validation, Writing – review & editing, Formal analysis, Methodology. AR-S: Formal analysis, Methodology, Supervision, Validation, Writing – review & editing. AC: Supervision, Validation, Writing – review & editing. MP: Supervision, Validation, Writing – review & editing. MM: Supervision, Validation, Writing – review & editing. EM: Supervision, Validation, Writing – review & editing. TJ: Supervision, Validation, Writing – review & editing. CC: Supervision, Validation, Writing – review & editing. SS-R: Supervision, Validation, Writing – review & editing, Conceptualization, Formal analysis, Funding acquisition, Methodology, Project administration, Writing – original draft.
